# Characterization of the interaction between Actinin-Associated LIM Protein (ALP) and the rod domain of α-actinin

**DOI:** 10.1186/1471-2121-10-22

**Published:** 2009-03-27

**Authors:** Tuula Klaavuniemi, Nanna Alho, Pirta Hotulainen, Annina Kelloniemi, Heli Havukainen, Perttu Permi, Sampo Mattila, Jari Ylänne

**Affiliations:** 1 Biocenter Oulu and Department of Biochemistry, PO Box 3000, University of Oulu, 90014 Oulu, Finland; 2 Department of Chemistry, PO Box 3000, University of Oulu, 90014 Oulu, Finland; 3 Institute of Biotechnology, PO Box 56, University of Helsinki, 00014 Helsinki, Finland; 4 Department of Pharmacology and Toxicology, PO Box 5000, University of Oulu, 90014 Oulu, Finland; 5 Department of Biological and Environmental Science and Nanoscience Center, University of Jyväskylä, PO Box 35, 40014 Jyväskylä, Finland

## Abstract

**Background:**

The PDZ-LIM proteins are a family of signalling adaptors that interact with the actin cross-linking protein, α-actinin, via their PDZ domains or via internal regions between the PDZ and LIM domains. Three of the PDZ-LIM proteins have a conserved 26-residue ZM motif in the internal region, but the structure of the internal region is unknown.

**Results:**

In this study, using circular dichroism and nuclear magnetic resonance (NMR), we showed that the ALP internal region (residues 107–273) was largely unfolded in solution, but was able to interact with the α-actinin rod domain *in vitro*, and to co-localize with α-actinin on stress fibres *in vivo*. NMR analysis revealed that the titration of ALP with the α-actinin rod domain induces stabilization of ALP. A synthetic peptide (residues 175–196) that contained the N-terminal half of the ZM motif was found to interact directly with the α-actinin rod domain in surface plasmon resonance (SPR) measurements. Short deletions at or before the ZM motif abrogated the localization of ALP to actin stress fibres.

**Conclusion:**

The internal region of ALP appeared to be largely unstructured but functional. The ZM motif defined part of the interaction surface between ALP and the α-actinin rod domain.

## Background

The muscle Z disc connects actin filaments from adjacent sarcomeres and is essential for force transmission and muscle integrity (for a recent review, see [1]). The main actin cross-linking protein in the Z disc is α-actinin, an antiparallel dimer. Each of the α-actinin monomers is composed of an N-terminal actin binding domain, a central rod region containing four spectrin repeats, and two pairs of EF-hands at the C-terminus (for review [2,3]).

**Figure 1:**
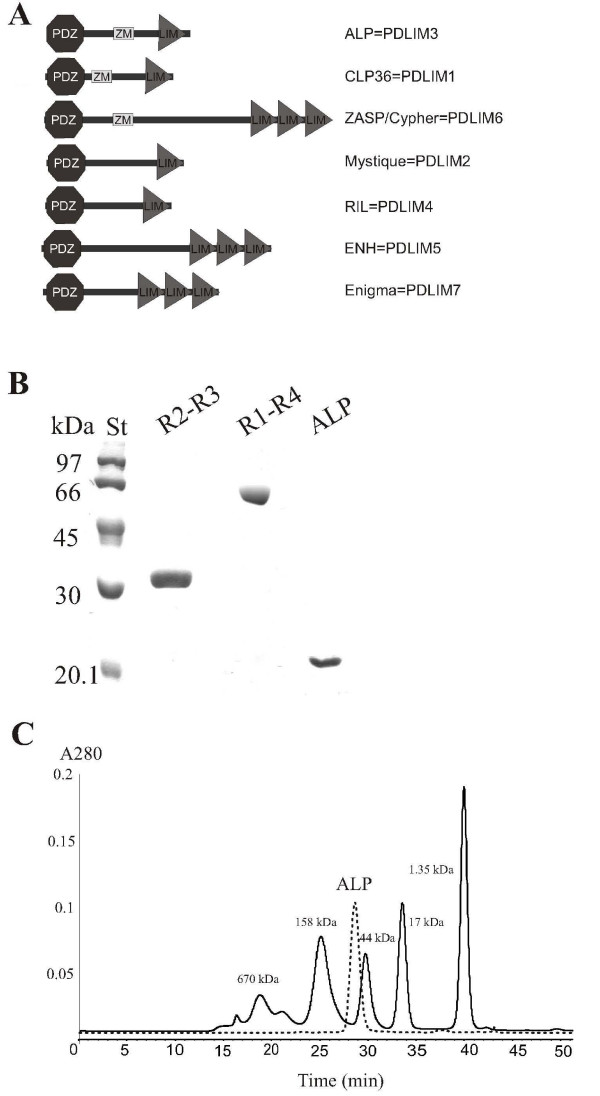
**Characterization of ALP107-273 protein**. A) Domain architecture of seven human PDZ_LIM proteins. B) SDS-PAGE of the proteins used in SPR measurements. R2-R3 denotes α-actinin spectrin repeats 2–3, R1-R4 spectrin repeats 1–4. ALP denotes the ALP107-273 fragment. Labelled ALP107-273 used in NMR experiments had a similar purity to the non-labelled form. C) Size exclusion chromatography showed that ALP107-273 eluted faster than expected based on its calculated monomer molecular weight (18 265 Da).

One group of α-actinin interacting Z disc proteins, the PDZ-LIM or ALP/Enigma family [4], is characterized by N-terminal PDZ and C-terminal LIM domains (Fig 1A). Of these family members, actinin-associated LIM protein (ALP), the Z band alternatively spliced PDZ protein (ZASP/Cypher), Enigma and Enigma homology protein (ENH) are predominantly expressed in muscle [5-12]. In contrast, the C-terminal LIM protein (CLP36) is expressed both in muscle and non-muscle cells [13-17], while reversion-induced LIM protein (RIL) and Mystique are expressed in non-muscle tissues only [17-21]. Inactivation of ALP and ZASP/Cypher genes in mice leads to cardiomyopathy and muscle dystrophy phenotypes [22,23]. In *Drosophila*, the single ALP/ZASP/Enigma gene is required for Z disc organization and for muscle attachment [24].

The PDZ domains of many, if not all, of these proteins interact with the C-terminal peptide of α-actinin [6,23,25-27]. In addition, ALP, ZASP/Cypher and CLP36 interact with the α-actinin rod domain [27-29] via sequences located between the PDZ and LIM domains, mapping close to a conserved 26 amino acid motif, the ZM motif, found in these three proteins [27,29]. Point mutations at or close to the ZM motif of ZASP/Cypher are found in cardiomyopathy patients [30,31], but we have been unable to detect a direct effect of these mutations on interaction with α-actinin [29].

The ZM motif is located in a sequence stretch of 200 or more residues between the PDZ and LIM domains of the ALP, ZASP/Cypher and CLP36, designated here as the internal region. Apart from the ZM motif and the recently found ALP-like motif [32], sequence diversity in the internal region is high, and in all family members there are areas 30–100 amino acid in length that are predicted to be unfolded by the program FoldIndex [33]. In this study, the aim was to structurally characterize the internal region of ALP and to further narrow its interaction site with the α-actinin rod region. The ALP internal region was found to be essentially a random coil in solution and interaction with the α-actinin rod could increase its stabilization. Furthermore, we found that a synthetic peptide covering part of the ZM motif interacted directly with the α-actinin rod.

## Results

### Expression and purification of ALP internal region fragments

**Figure 2:**
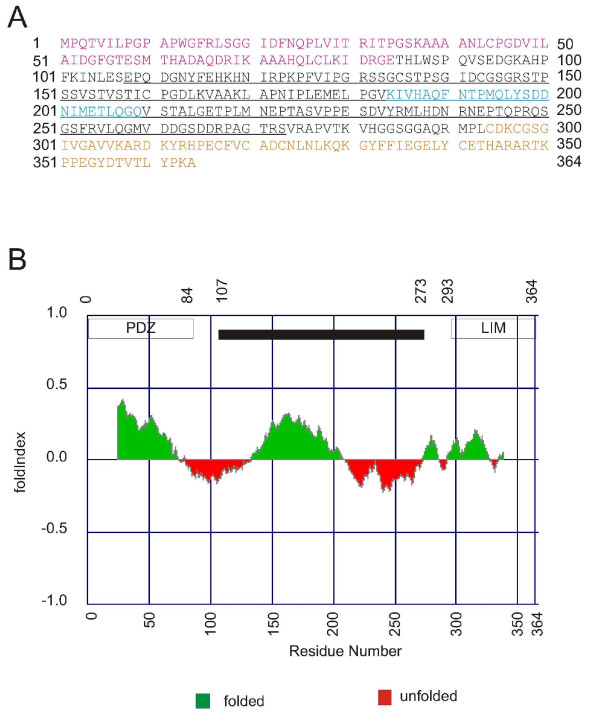
**Amino acid sequence analysis**. A) The full sequence of human ALP is shown. The PDZ domain is shown in red, the ZM motif in blue and the LIM domain in yellow. The 107–273 fragment used in this study is underlined. B) Analysis of ALP with the Foldindex server . Predicted folded areas are indicated in green and unfolded areas in red. The boxes above the graph indicate the PDZ and LIM domains as well as the 107–273 fragment.

As the internal region of ALP is involved in localization of the protein and interaction with α-actinin [27], it was of great interest to study the structure and function of this region in more detail. According to the SMART server [34], the PDZ domain of human ALP ends at amino acid residue 83 while the LIM domain begins at residue 294. In our previous studies [27], we used the fragment containing residues 112–284, but found that during purification this fragment often degraded. For this reason, we tried to find optimal protein fragments of the ALP internal region and we tested several different constructs around the area that had sequence features characteristic for folded domains when predicted with servers like Foldindex [33] (Fig 2) and DisEMBL [35]. We found that a 167 residue construct (ALP107-273) was optimally stable, and could be purified in high amounts and concentrated up to 30 mg/ml. SDS-PAGE analysis of this fragment revealed a single protein band of the expected molecular weight of 18265 Da (Fig 1B). The molecular weight of ALP107-273 was also verified by MALDI-TOF mass spectrometry. In gel filtration chromatography, the fragment eluted as a single peak immediately prior to the globular protein standard of 44 000 Da (Fig 1C), suggesting that it was either a very elongated monomer, dimer or trimer.

### Functional tests of ALP107-273

**Figure 3:**
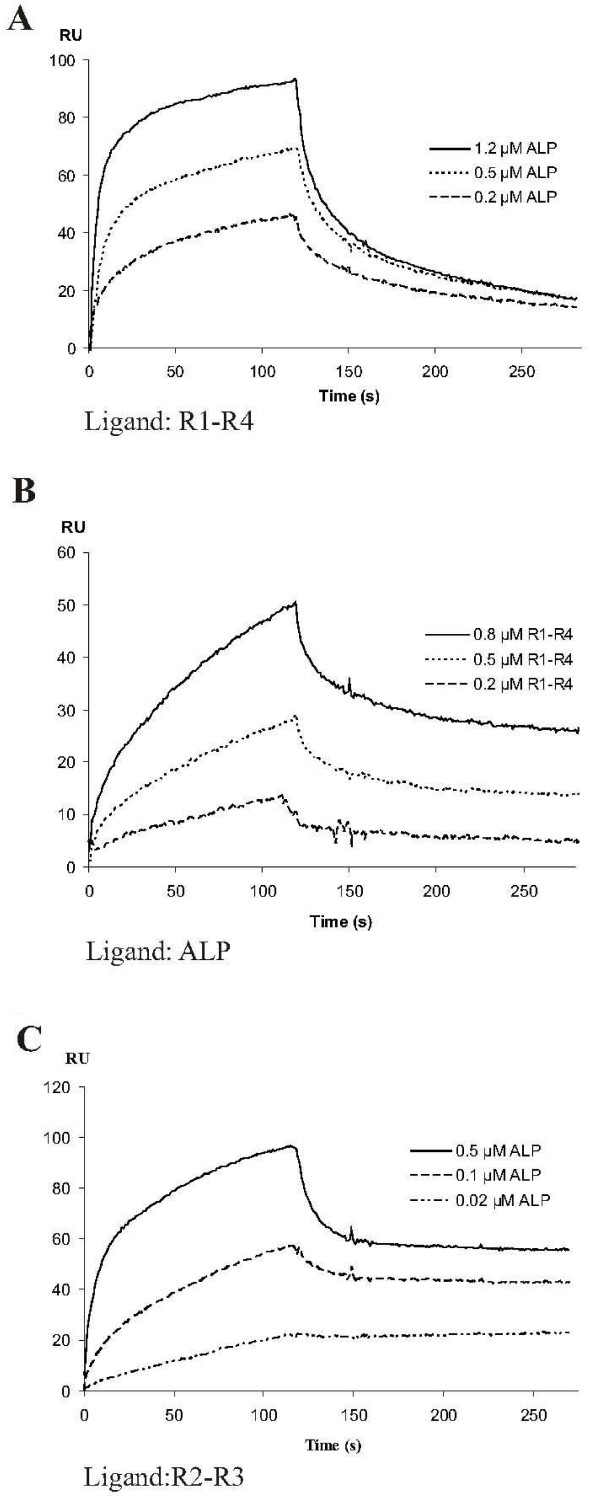
**SPR measurements of ALP107-273/α-actinin interaction**. A) ALP107-273 as the soluble analyte with immobilized α-actinin spectrin repeats 1–4 (R1-R4). B) R1-R4 interaction with immobilized ALP107-273. C) ALP107-273 interaction with immobilized α-actinin spectrin repeats 2–3 (R2-R3).

To test whether ALP107-273 was functional, we measured the interaction of the purified protein fragment with α-actinin by surface plasmon resonance (SPR) and tested the localization of a yellow fluorescent protein (YFP) fusion protein construct in living cells. The SPR measurements were made either by immobilizing ALP107-273 on the chip, using the α-actinin rod fragments as a soluble analyte, or the other way around. A clear interaction was observed in both experimental setups (Figs 3A and 3B) and the apparent dissociation equilibrium constants (K_d_) were in the low micromolar range. When an α-actinin rod fragment consisting of four spectrin repeats (R1-R4) was immobilized and ALP107-273 used as the analyte, the K_d _was 2.7 × 10^-7 ^M. With immobilized ALP107-273 and soluble R1-R4, the K_d _was 4.5 × 10^-6 ^M. These values are in the same range as our previous measurements that used longer ALP fragments [27]. ALP107-273 interacted with a shorter piece of the α-actinin rod region that contained two spectrin repeats (R2-R3) with an apparently higher affinity (Kd = 6.8 × 10^-9^) than it did with R1-R4 (Fig 3C).

**Figure 4:**
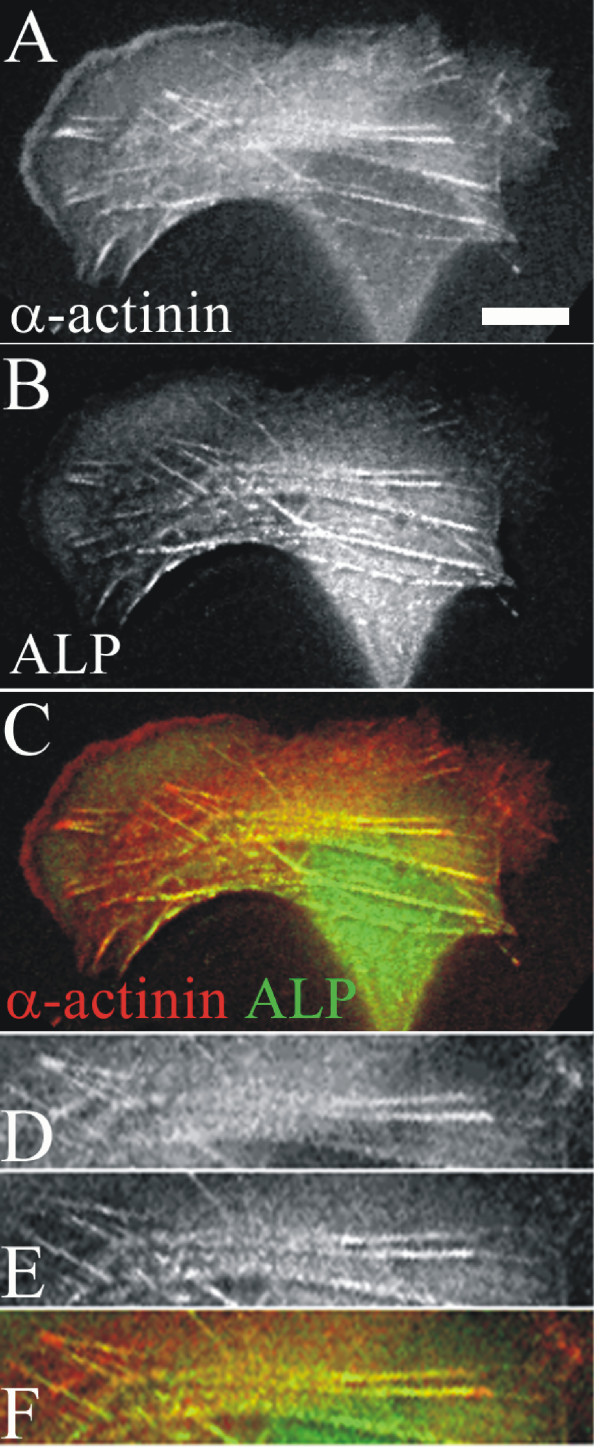
**Localization of ALP107-273 in living cells**. Fluorescence micrograph of a U2OS cell expressing α-actinin-CFP (A and red in C) and YFP-ALP107-273 (B and green in C). A higher magnification view of a region containing stress fibres is displayed in the bottom three panels (D, E, F). Bar 10 μm.

The YFP-fusion construct of ALP107-273 was expressed in U2OS cells together with the α-actinin cyan fluorescent protein (CFP). In these cells, α-actinin localizes to the leading lamellipodia, to focal adhesions and to actin stress fibres. In contractile stress fibres, α-actinin exhibits a punctate pattern (Fig 4A, D and red in 4C, F). ALP107-273 co-localized with α-actinin in the stress fibres, but no co-localization was seen in either the cell edge or the focal adhesions (Fig 4B, E and green in 4C, F).

Taken together, the SRP interaction measurements and live cell experiments using YFP fusion protein indicated that ALP107-273 interaction with α-actinin was similar to the full length ALP [27].

### Structural characterization of ALP107-273

**Figure 5:**
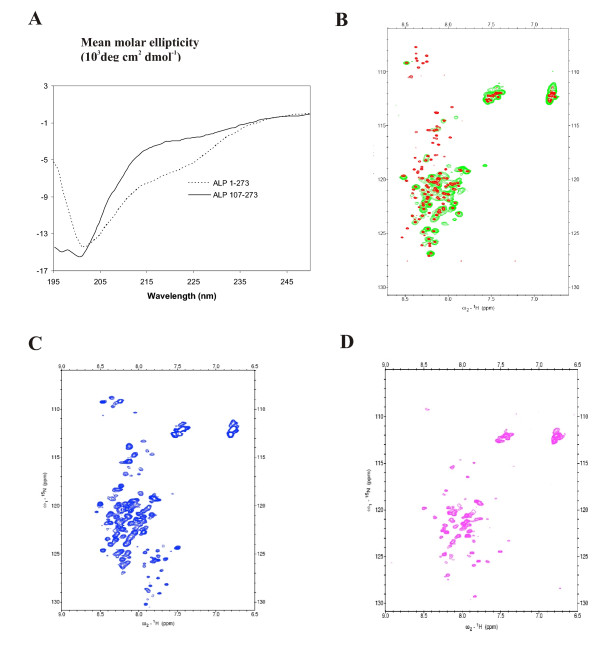
**Structural analysis of ALP107-273**. A) The CD spectrum of ALP107-273 is consistent with a random coil conformation, whereas the PDZ domain containing ALP1-273 fragment has some β-strand features. B) Overlaid ^15^N HSQC NMR spectra of 1.6 mM ALP107-273 in the purification buffer (green) and after addition of 40 mM Asp (red). C) ^15^N-HSQC spectrum of 84 μM ALP107-273 in the presence of 218 μM unlabelled R1-R4. D) Identical spectra as in C taken from 80 μM μM ALP107-273 alone. The low intensity signals in the lower right part of the spectra in C and D are observed for ALP concentrations under 100 μM, and apparently represent another conformation of ALP. Owing to low sample concentration, no attempt was made to assign these signals.

To study the structure of ALP107-273 we utilized circular dichroism (CD) and nuclear magnetic resonance (NMR) spectroscopy. The CD spectrum suggested that ALP107-273 is mostly unfolded (Fig 5A). No features characteristic of either α-helical or β-sheet structures were seen. In comparison, the CD spectrum of ALP1-273 showed a slight increase in β-strand content that might be attributed to the PDZ domain (Fig 5A).

**Figure 6:**
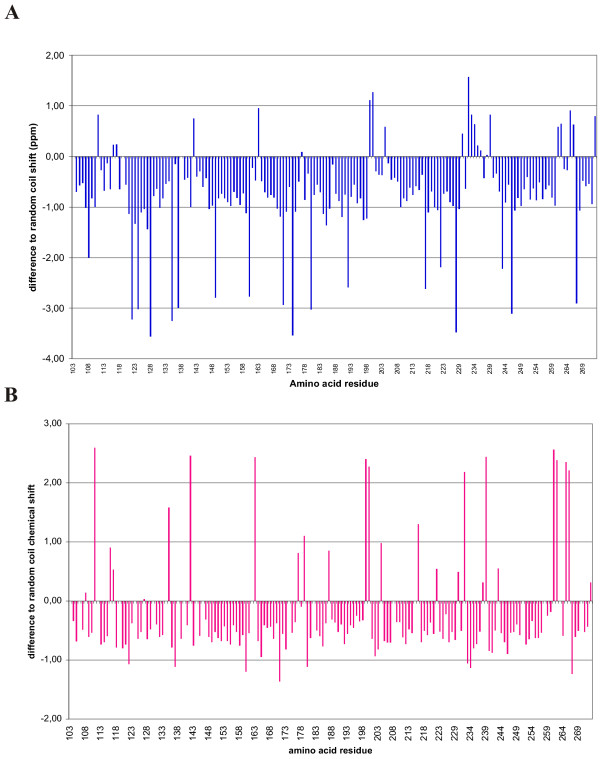
**Comparison of ALP107-273 NMR chemical shifts with those observed in random coil conformations**. A) Difference of ^13^C Cα chemical shifts with random coil as a function of amino acid residue number. The random coil values were taken from the Biological Magnetic Resonance Data Bank . The average difference for ^13^C Cα chemical shifts in an α-helix is +3 and in a β sheet is -1.5 [39]. B) The difference in ^13^C Cβ chemical shift to random coil conformation. The average reference value is -1 in an α helix and +3 in a β sheet [39]. Based on this analysis ALP107-273 does not appear to contain sequential residues with typical α helix or β sheet conformations.

The first ^15^N-HSQC spectrum measured in normal buffer conditions from uniformly ^15^N-labeled ALP107-273 was in line with the CD measurements as it showed the collapsed spectrum characteristic of an unfolded protein (Fig 5B, green). However, we were able to partially stabilize the structure by adding 40 mM Aspartic acid pH 4.0 (Fig. 5B, red) [36,37]. Under these conditions, iHNCA, HNCA, HNCACO, HNCO, HNCOCA, HNCACB and CBCA(CO)NH spectra were acquired and used for backbone assignment [38]. The ALP107-273 fragment contained 167 residues, of which 16 were prolines. All together, 147 of the 151 non-proline residues could be assigned. When the ^13^C chemical shift values of all assigned Cα and Cβ atoms were compared to the average value of the corresponding residues in random coil conformation [39], no clear indications of either α helix or β sheet secondary structures were observed (Fig 6). Thus, NMR analysis indicated that ALP107-273 was mostly unstructured with no secondary structure.

The interaction with the α-actinin rod region could have induced some structural changes in ALP107-273. Assessing this by titration was complicated because the complex between ALP107-273 and the large α-actinin rod dimer of 114 kDa was undetectable by NMR. To be able to measure the spectrum of an unbound ALP107-273 that is in dynamic equilibrium between α-actinin bound and unbound states, we used a rather low concentration of ALP107-273 and observed increasing levels of ALP107-273 ^15^N-HSQC peaks upon addition of α-actinin (Fig 5C). Most of the peaks (110, or 75%) could be correlated with those assigned in the presence of 40 mM Aspartic acid. Only a minor fraction of the peaks were induced by ALP dilution alone (Fig 5D). The NMR titration studies suggested that structure of ALP107-273 was partially stabilized by the interaction with the α-actinin rod region.

### Mapping of the interaction site with synthetic peptides and deletions

**Figure 7:**
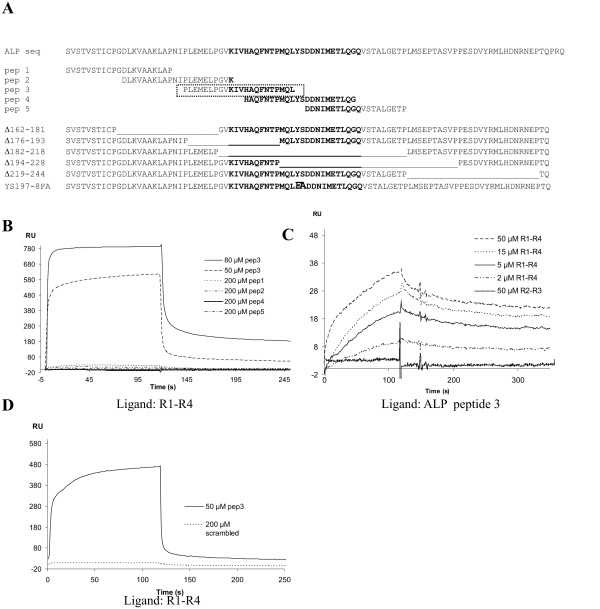
**SPR analysis of peptide interactions**. A) The ALP sequence between residues 152 and 246 with the ZM consensus motif marked in bold. Sequences of five overlapping peptides of ALP internal region are shown next, followed by the five deletion mutants and one point mutant. B) SPR sensograms showing that ALP peptide 3 interacts with the rod domain, whereas peptides 1, 2, 4 and 5 do not. R1-R4 was used as a ligand. C) R1-R4 interaction with ALP peptide 3 (used as a ligand) is concentration-dependent, but R2-R3 does not interact with peptide 3. D) Comparison between the binding of peptide 3 and its scrambled version containing the same amino acids in random order.

As NMR studies suggested that ALP107-273 could exist in an elongated, unfolded conformation, even when interacting with α-actinin, we chose to map the interaction site using overlapping synthetic peptides (20–22 residues long). We focused our efforts on the minimum interaction area mapped by truncation mutagenesis, namely ALP residues 151–232, which included the ZM motif [27]. The sequences of these peptides are shown in figure 7A. Only peptide 3 (PLEMELPGVKIVHAQFNTPMQL, corresponding to residues 175–196) bound to the immobilized α-actinin rod domain (composed of spectrin repeats R1-R4) in the SPR experiments. A clear interaction was seen with 50 μM and 80 μM peptide concentrations, whereas the other four peptides failed to interact even at 200 μM concentrations (Fig 7B). To verify the interaction of peptide 3 with the rod, peptide 3 was immobilized and the rod was used as the soluble analyte (Fig 7C). The rod interacted with the peptide with apparent K_d _of 3.0 × 10^-6 ^M. However, in contrast to the ALP107-273 fragment, peptide 3 did not interact with R2-R3 (Fig 7C). A scrambled version of peptide 3 (MNKPEQALLVQIHPLVTEPFMG) did not interact with the rod domain, verifying the sequence specificity of the interaction with the rod (Fig 7D). Thus, an area of residues 175–196 containing the N-terminal half of the ZM motif of ALP appeared to interact directly with the α-actinin rod domain, but not with its two central spectrin repeats, R2-R3.

**Figure 8:**
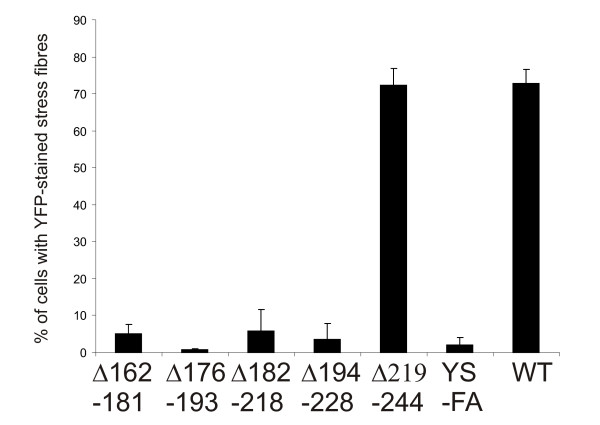
**Deletion mutations at the ZM region disrupt ALP107-273 localization**. The percentages of cells with YFP localization at stress fibres. YFP was fused to ALP107-273 without any modifications (WT) or with deletions or mutations (deletions and mutations shown in graph). Note that four deletion mutants and the YS197-198FA point mutation (YS-FA) abrogated localization. In cells expressing YFP alone, < 1% were scored positive.

The peptide binding studies suggested that sequences before and at the ZM motif of ALP are sufficient for interaction with α-actinin. To further test if this region was really necessary for the interaction, we constructed five overlapping deletions in ALP107-273 between residues 162 and 244. The deletions were 18–37 amino acids long and each was designed to include a stretch between two proline residues (Fig 7A). Four deletions that overlapped with peptide 3 totally abrogated the ability of ALP107-273 to localize at stress fibres (Fig 8), whereas a deletion after the ZM motif did not affect the localization. As the ZM motif contains conserved tyrosine and serine residues that might be phosphorylated, we also made point mutations where these residues were changed to phenylalanine and alanine, respectively. This mutant also failed to localize to stress fibres (Fig 6). Taken together, the peptide binding studies and the mutant studies suggested that the ZM motif is sufficient and necessary for ALP interaction with α-actinin in the stress fibres.

## Discussion

α-Actinin interacting PDZ-LIM proteins are important for sarcomeric integrity as well as being involved in hypertrophic stretch activated signalling pathways in the muscle Z disc (for reviews, see [40-43] via their interactions with protein kinase C isoforms and calsarcins [9,22,23,30,31,44,45]. Although the PDZ and LIM domains are well-known protein folds, the functional and structural properties of the internal regions in this family of proteins are still poorly characterized.

In previous studies, we showed that ALP, CLP36 and ZASP/Cypher, which all have a ZM motif in the internal region, interacted with the α-actinin rod and that the ZM motif (ALP residues 184–209) might be important for this interaction [27,29]. In the current paper, we have been able to map the interaction site in more detail. We showed that a synthetic peptide spanning ALP residues 175–196 was able to interact directly with the α-actinin rod. Two other peptides spanning parts of the ZM motif did not interact and neither did two peptides before the ZM motif. On the other hand, several deletion mutants before or at the ZM motif abrogated the localization of YFP-ALP fragments to α-actinin containing structures. Notably, a deletion after the ZM motif had no effect. A mutation at the conserved Tyr-Ser sequence in the ZM motif (residues 197–198) also abrogated localization of ALP in cells. Further studies are required to test whether this effect is dependent on phosphorylation, and whether phosphorylation could explain the lack of ALP in focal adhesions where α-actinin is located.

Thus, our peptide binding studies and deletion mutations suggest that the ZM motif is directly involved in the interaction with the α-actinin rod. However, these results do not rule out the involvement of other areas of the ALP internal region in the interaction. On the contrary, the involvement of other areas was suggested by the finding that while peptide 3 interacted strongly with the α-actinin rod fragment composed of four spectrin repeats (R1-R4), it did not interact with the two central spectrin repeats (R2-R3) of α-actinin. The ALP107-273 fragment, on the other hand, interacted with both R1-R4 and R2-R3 fragments. These data are compatible with a hypothesis that the ALP internal region may have an extended interaction surface on α-actinin and that the interaction surface may cover a large area of the α-actinin rod domain.

Structural analysis showed that the ALP internal region is largely unstructured and that interaction with α-actinin partially stabilizes its conformation, but does not induce any detectable secondary structure elements. We showed previously that two ALP molecules can interact with the dimeric α-actinin rod domain [27]. Combining this information with our current structural and hydrodynamic analyses suggests that the ALP internal region is an elongated, flexible monomer in solution, although we cannot exclude the formation of dimer. Notably, despite a lack of ordered structure, this fragment is rather stable and it can be expressed, purified and concentrated to a high degree. Based on sequence analysis by programs such as FoldIndex [33] or DisEMBL [35], the ALP internal region cannot be classified as being intrinsically unfolded (Fig 2). Analysis of the corresponding regions of other PDZ-LIM family members with the same programs yielded quite similar results (data not shown).

## Conclusion

Our current data are compatible with the hypothesis that the ALP internal region exists in an open, flexible conformation and forms a long interaction surface with the α-actinin rod region. We have also shown that sequences both before and at the conserved ZM motif are required for interaction with α-actinin and that a short peptide from this area can interact directly.

## Methods

### Constructs used

Internal fragments of human ALP (**AF039018**) and chicken ALP (**AJ249218**) were cloned into a modified pET24d vector (Novagen, Merck Biosciences, Schwalbach, Germany) as described earlier [27] using BsmBI (NcoI)- NotI cloning sites. Rod fragments containing spectrin repeats 1–4 (residues 274–746) or repeats 2 and 3 (residues 371–637) of human α-actinin 2 have been described previously [46,47]. A YFP-fusion of ALP107-273 was generated in a pEYFP-C1 vector (Clontech, BD Biosciences) at an EcoRI-BamHI restriction site. The deletion constructs of pEYFP-ALP107-273 were generated by PCR. The α-Actinin-CFP construct is described elsewhere [48]. All constructs were verified by DNA sequencing.

### Protein expression and purification

ALP internal fragments and an α-actinin rod containing the spectrin repeats 1–4 (R1-R4) and a fragment containing spectrin repeats 2 and 3 (R2-R3) were expressed at +37°C for 3–4 h in *Escherichia coli *BL21(DE3) strain as previously described [27]. Briefly, proteins were purified using nickel nitrilotriacetic acid-agarose (Qiagen) as a first step. The His-tag was removed by tobacco etch virus protease (Invitrogen). For further purification, size exclusion chromatography (Superdex 75 16/60 and 26/60 columns, Amersham Biosciences) for ALP, and ion exchange (ProteinPakQ 8HR columns, Waters) for α-actinin fragments were used. The molecular weight standards for the gel filtration were obtained from Bio-Rad Laboratories. For NMR analysis, ^15^N-labelled ALP107-273 and ^15^N^13^C-labelled ALP107-273 were produced in general M9-media with supplements [49]. Expression was induced with 1 mM isopropyl-1-thio-β-D-galactopyranoside (IPTG) at +37°C for 8 hrs in *E. coli *BL21(DE3) strain. Low molecular weight markers used in SDS-polyacrylamide electrophoresis were obtained from Amersham Biosciences. Five ALP peptides were purchased from EZBiolab Inc. (Westfield, IN, USA).

### Circular dichroism

A JASCO J-715 spectrometer (JASCO Corporation, Hachioji City, Tokyo, Japan) was used for circular dichroism measurements. Purified ALP proteins at a concentration of approximately 30 mg/ml were diluted to 0.15 mg/ml with 20 mM Tris, pH 8 buffer. The far-UV spectrum (195–250 nm) was measured at 20°C and the background effect of buffer was subtracted. The instrument settings were as follows: response 1 sec, scan speed 50 nm/min, cell length 0.1 mm, number of scans 16.

### Localization studies of ALP mutants

The pEYFP ALP107-273 and mutants were transfected into CHO cells as described previously [27]. Following 24 hrs transfection, the cells were seeded for 4 hrs on coverslips coated with 10 μg/ml human plasma fibronectin (Sigma) and fixed with 4% paraformaldehyde in 140 mM NaCl, 10 mM Na-Phosphate pH 7.4. The coverslips were coded and the number of cells with YFP localization at stress fibres was counted by two observers who were blinded to the identity of the samples. At least 100 cells were scored from each coverslip, four coverslips were used per transfection, and the experiment was repeated at least three times.

### Live cell microscopy

Human osteosarcoma (U2OS) cells were maintained in Dulbecco's modified Eagle's medium (DMEM) supplemented with 10% fetal bovine serum (Hyclone), 2 mM L-glutamine, penicillin and streptomycin (Sigma-Aldrich). Transfected cells were re-plated on fibronectin (10 μg/ml) coated glass bottom dishes (MatTek). General growth medium was used as imaging medium. The time lapse images were acquired with an IX-71 inverted microscope (Olympus) equipped with a Polychrome IV monochromator (TILL Photonics) with the appropriate filters, an Andor iXon (Andor) camera, a heated sample environment (+37°C) and CO_2_-control. A PLAPON 60xO TIRFM 60x/1,45 (oil) objective (Olympus) was used. Software for image acquisition was TILL Vision version 4 (TILL Photonics).

### Nuclear magnetic resonance

After defrosting 100 μl of the purified ^15^N labelled ALP (1.6 mM) in buffer solution (20 mM TRIS, 150 mM NaCl, 1 mM EDTA and 1 mM DTT, pH 8), a NMR sample of pure ALP was prepared by adding 10% D_2_O to the defrosted ALP and transferring the sample to a susceptibility matched Shigemi tube (*Shigemi Inc*., tube matched to water with 8 mm bottom). Initially, a ^15^N-HSQC spectrum of ALP107-273 was acquired as a reference spectrum. The titration series was initiated by preparing a sample containing 45 nmol ALP, 15 nmol rod domain of α-actinin (R1-R4, 277 μM and 236 μM in 20 mM TRIS and 50 mM NaCl, pH 8), 15 μl buffer solution of R1-R4 and 10% D_2_O to a susceptibility matched Shigemi tube. A ^15^N-HSQC spectrum was measured and the titration was continued in a stepwise manner by adding 15 nmol of R1-R4 to the sample and acquiring a new ^15^N-HSQC spectrum at each titration point, until the ratio of 3:8 (ALP:R1-R4) was reached. As a control, titration of ALP was performed with R1-R4 buffer (20 mM TRIS and 50 mM NaCl, pH 8). In order to enable direct comparison of the ^15^N HSQC spectra at each data point, identical experimental and processing parameters were employed. Measurements were performed with a Bruker Avance DRX 500 MHz spectrometer (Bruker BioSciences, Billerica, Massachusetts, USA) and a 5 mm triple resonance inverse probehead at room temperature. Measurement of ^15^N/^13^C enriched ALP was performed with a Varian Unity INOVA 800 MHz spectrometer (Varian, Palo Alto, California).

### Surface plasmon resonance

A Biacore 3000 system (Biacore, Uppsala, Sweden) was used for surface plasmon resonance (SPR) analysis. Ligand immobilization was performed via amine coupling to gold sensor chips (CM5). The running buffer was 20 mM Tris, pH 7.4, 150 mM NaCl, 0.005% surfactant P20 (BR-1000-54, Biacore AB, Uppsala, Sweden).

## Authors' contributions

TK planned the study together with JY, made the DNA constructs for protein expression and localization, purified the proteins, performed all of the biochemical experiments and wrote the article. AK participated in DNA cloning and protein expression. NA made the NMR measurements and did the analysis together with PP and SM. PH performed the time lapse microscopy experiment. HH made and analyzed the ALP deletion GFP constructs. JY supervised the study, analyzed the data and wrote the article. All authors participated in the writing and have read and approved the final manuscript.
